# The Effect of Cycloplegia on Ocular Alignment and AC/A Ratio

**DOI:** 10.18502/jovr.v19i1.15442

**Published:** 2024-03-14

**Authors:** Amir Asharlous, Hassan Hashemi, Abbasali Yekta, Alireza Riazifar, Asgar Doostdar, Mahsa Sadri, Amir Rakhshan, Hadi Ostadimoghaddam, Mehdi Khabazkhoob

**Affiliations:** ^1^Noor Ophthalmology Research Center, Noor Eye Hospital, Tehran, Iran; ^2^Department of Optometry, Mashhad University of Medical Sciences, Mashhad, Iran; ^3^Department of Optometry, School of Rehabilitation Sciences, Iran University of Medical Sciences, Tehran, Iran; ^4^Noor Research Center for Ophthalmic Epidemiology, Noor Eye Hospital, Tehran; ^5^Department of Foreign Languages, Tehran University of Medical Sciences, Tehran, Iran; ^6^Refractive Errors Research Center, Mashhad University of Medical Sciences, Mashhad, Iran; ^7^Department of Basic Sciences, School of Nursing and Midwifery, Shahid Beheshti University of Medical Sciences, Tehran, Iran

**Keywords:** Convergence, Mydriatics, Strabismus

## Abstract

**Purpose:**

The present study sets out to investigate the effect of cyclopentolate-induced cycloplegia on distance and near deviation and the accommodative convergence/accommodation (AC/A) ratio.

**Methods:**

This prospective study was performed on 30 subjects. The inclusion criteria included a lack of any active ocular pathology and systemic diseases, no history of ocular surgery, and nonuse of various medications. Refraction, near and distance deviation were measured for all subjects, and the same examinations were repeated after the administration of two drops of cyclopentolate 1% to both eyes.

**Results:**

The obtained data from 30 subjects, including 19 males, with a mean age of 22.53 ± 1.74 years were analyzed. The mean ± SD of near deviation in dry and cycloplegic conditions were –6.9 ± 8.1 and +6.4 ± 9.1 prism diopters, respectively, which were statistically significant (*P* < 0.001). Distance deviation in cycloplegic conditions demonstrated an average difference of 0.8 prism diopters, compared to dry conditions (*P* < 0.001). AC/A ratios were 4.7 ± 2.5 and 9.7 ± 3.9 (Δ/D) in non-cycloplegic and cycloplegic conditions, respectively, which was a statistically significant difference (*P* < 0.001). The multiple regression indicated that among all under study variables, refraction (B coefficient: –2.4; *P* < 0.001) and near pre-cycloplegic deviation (B coefficient: 0.56; *P* < 0.001) were significantly associated with post-cycloplegic near deviation.

**Conclusion:**

The results of this study indicated that cycloplegia causes a considerable esophoric shift in near deviation and a negligible esophoric shift in distance deviation. As a result, the AC/A ratio demonstrated a significant increase due to unequal changes in near and distance deviation.

##  INTRODUCTION

Cycloplegic refraction is an indispensable part of most ocular examinations, especially in binocular vision and epidemiological studies.^[1]^ The use of anticholinergic (cycloplegic) eye drops inhibits the action of muscarinic acetylcholine receptors in the ciliary body and hence prevents the contraction of ciliary muscles.^[2]^ In fact, these agents cause a local accommodation paralysis and are used to increase the accuracy of refraction and discover the latent components of refractive error. This is of special importance in some patients, especially the hyperopic cases.^[3,4]^

There are several cycloplegic agents, including atropine, homatropine, cyclopentolate, and tropicamide.^[5]^ In recent years, cyclopentolate 1% has been introduced as the gold standard for cycloplegic refraction in the majority of studies. ${\;}^{\left[1,6\right]}$ This agent is efficient for most routine refractive examinations and induces adequate accommodation paralysis.^[6]^

The accommodation reflex occurs through a near triad response along with convergence and pupillary miosis, and this association is clinically important.^[7]^ The relationship between accommodation and convergence depends on the AC/A ratio and is diagnostically and therapeutically important in the field of binocular vision and considered a major factor in the classification of different types of strabismus and in the selection of treatment methods.^[8]^ Accommodation paralysis by the instillation of cycloplegic drops raises the following question: What would be the ocular alignment in the case of inhibition of ciliary muscle cholinergic receptors, given that simultaneous impulses are also transmitted to medial rectus muscles for convergence?

Jeong et al investigated the state of near deviation after instilling a combination of tropicamide and phenylephrine drops in hyperopic children with esotropia. They reported that the use of these agents increased near esotropia.^[9]^ The referred study did not assess distance deviation and AC/A changes, and it was merely performed on children with hyperopia and esotropia. The effect of cycloplegia on far and near deviation is still unclear, especially in individuals without hyperopia and esotropia. Moreover, there is no information about changes in the AC/A ratio under cycloplegic conditions. The present study aimed to fully examine ocular alignment and AC/A ratio under cycloplegic conditions.

##  METHODS

### Study Participants 

The present study was conducted on 30 students (including 19 males) within the age range of 20–27 years from the Faculty of Rehabilitation, Iran University of Medical Sciences in 2020. We conducted this study on 30 students only, as this study was performed under COVID-19 pandemic conditions and cycloplegic instillation also caused discomfort for some patients. The inclusion criteria were best-corrected visual acuity of 20/20, no history of any ocular surgery, especially cataract and strabismus surgery, nonuse of ophthalmic and systemic medications, absence of any ocular surface inflammation, and absence of corneal or crystalline lens opacity. The ethical considerations of the current study were reviewed by the Ethics Committee of Iran University of Medical Sciences and were approved (ethics code: IR.IUMS.REC.1399.146). This study was performed in accordance with the tenets of the Declaration of Helsinki and informed written consent was obtained from all participants prior to enrollment.

In the beginning of the study, a complete systemic and ocular history was taken from each individual; moreover, complete visual acuity and slit-lamp examinations were performed to ensure meeting the inclusion criteria. Next, the pupillary distance was measured using a spotlight and a ruler. The refractive status was evaluated with an auto-refractometer (HUVITZ, HRK8000a, Korea), and the results were refined by retinoscopy and recorded in the participants' medical records. Binocular alignment (corrected with full correction glasses) was accurately evaluated using prism and alternate cover test at distance (6 m) and near (accommodative target at 40 cm). Two drops of cyclopentolate 1% were instilled in both eyes in 5-min intervals, and the examination was repeated 30 min after the second drop. Firstly, the cycloplegic refraction was performed with an auto-refractometer and retinoscope; then, the deviation was measured (with the exact correction of cycloplegic refractive error). The prism alternate cover test was performed at a distance of 6 m and 40 cm, and the exact amount of phoria was measured and recorded. Furthermore, in all subjects, near phoria was examined with a refractive correction along with a +2.5 addition, and the exact amount of phoria was measured and recorded. All examinations were performed by one examiner only, using the same accommodative target, in the same place, and with the same illumination status before and after cycloplegia instillation in each session. These examinations were measured three times and the mean was recorded for each type of measurement.

### Statistical Analysis 

The obtained data were analyzed in the SPSS software (IBM Corporation, USA), and descriptive statistics indices were calculated for all variables. The AC/A variable before and after drop instillation was calculated based on the values of distance and near deviation considering FPD using the following formula: AC/A = FPD + 0.4 (D ${\;}_{n}$ – D ${\;}_{f}$ ),^[10]^ in which, FPD (far pupillary distance) was presented in centimeters. D ${\;}_{n}$ and D ${\;}_{f}$ were the values of near and distance deviation, respectively, and were presented in the prism diopter unit. Esophoria values were represented with positive and exophoria values with negative signs.

The gradient method was not used in the present study since the subjects were in cycloplegic conditions. As positive and negative lenses are used in the gradient method, this would not be practical under cycloplegia.

Data distribution normality was evaluated using Kurtosis and Skewness indices, as well as the Kolmogorov–Smirnov statistical test. These assessments revealed that the majority of the studied data were not normally distributed; therefore, non-parametric tests were used to compare and examine the correlations. Wilcoxon signed-rank test was used to compare before–after, and the Spearman correlation test was utilized to examine the correlation of variables.

As the post-cycloplegic deviation data were normally distributed, multiple regression model was used to investigate the effect of different variables on the degree of post-cycloplegic deviation. In all analyses of the present study, *P*-values < 0.05 were considered statistically significant.

##  RESULTS

The data obtained from 30 subjects (mean age: 22.53 ± 1.74 years, 19 males) were analyzed. Table 1 displays the descriptive indices of the studied variables prior to and after cycloplegia, along with the *P*-values.

As illustrated in this table, the use of cycloplegic drops caused an average change of 13.3, 0.8, and 5.0 prism diopters in near deviation, distance deviation, and AC/A ratio, respectively. In addition, a statistical comparison was made between the magnitude of near deviation in cycloplegic conditions both with and without a +2.5 addition.

According to the table, the near deviation had a difference of 11.5 prism diopters in these two conditions, and this difference was statistically significant (*P* < 0.001). Moreover, the size of near deviation in pre-cycloplegic conditions was compared with near deviation in post-cycloplegic with a +2.5 addition. As shown in Table 1, these two conditions had a difference of 1.8 prism diopters, which was statistically significant (*P* = 0.001).

Multiple regression analysis was used to assess the effects of all studied parameters, including age, AC/A before cycloplegia, refraction, size of distance deviation, size of near deviation, differences between near and distance deviation before cycloplegia, and the refraction difference before and after cycloplegia, on the change of near deviation and the magnitude of near deviation after cycloplegic refraction. Among all these parameters, the magnitude of near deviation after cycloplegic refraction was only correlated with refraction (B coefficient: –2.4, *P* < 0.0001) and the magnitude of near deviation (B coefficient: 0.59, *P* < 0.0001). The other variables did not show a significant relationship.

**Figure 1:**
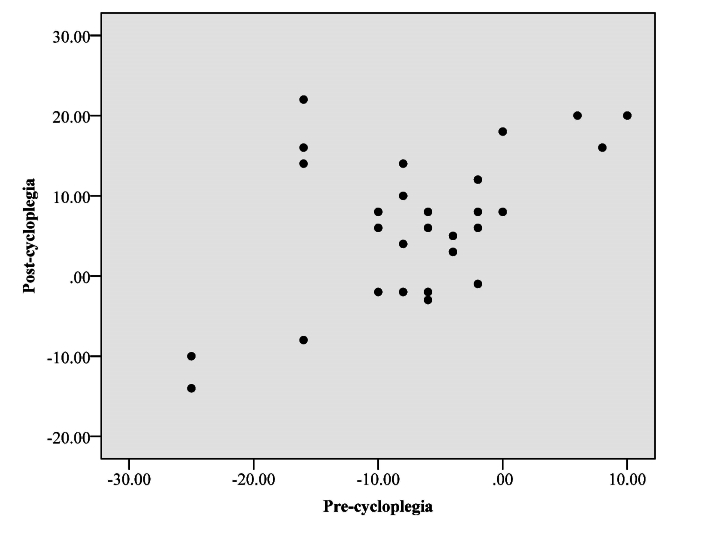
The correlation of near deviation before and after cycloplegia.

**Table 1:** Mean and standard deviation of the studied variables prior to and after cycloplegia.


	**Pre-cycloplegia (Mean ± SD)**	**Post-cycloplegia (Mean ± SD)**	***P*** **-value**
SE.OD (D)	–1.8 ± 2.1	–1.4 ± 2.1	< 0.001
SE.OS (D)	–1.8 ± 2.1	–1.4 ± 2.2	< 0.001
D ${\;}_{n}$ (Δ)	–6.9 ± 8.1	+6.4 ± 9.1	< 0.001
D ${\;}_{f}$ (Δ)	–2.8 ± 4.7	–1.9 ± 4.5	< 0.001
D ${\;}_{N2.5}$ (Δ)	**–**	–5.1 ± 7.4	–
AC/A (Δ/D)	4.7 ± 2.5	9.7 ± 3.9	< 0.001
	
	
SD, standard deviation; SE, spherical equivalent; OD, oculus dexter; OS, oculus sinister; D, diopter; D ${\;}_{n}{\;}_{,}$ near deviation with +2.50 addition; AC/A, accommodative convergence/accommodation			

Figure 1 illustrates the correlation of near deviation before and after cycloplegia in each participant. As shown in this figure, an esophoric shift was clearly observable in almost all participants, however, the magnitude of esoshift was different according to each patient's binocular condition. In some patients with more near exodeviation, we observed a remarkable esophoric shift after cycloplegia, which may show more binocular instability in these cases. Of course, this should be investigated in other studies.

##  DISCUSSION

The current research, to the best of our knowledge, is one of the few studies conducted on ocular misalignment in near and distance fixation, as well as changes in the AC/A ratio with and without cycloplegia. The results of the present study can be helpful in making clinical diagnosis and discovering new therapeutic strategies to control the deviations. These results will also help to enrich the existing body of literature on the topics discussed, expanding the reference for future studies.

Cyclopentolate-induced cycloplegia caused a significant esophoric shift in near deviation. In line with the present research, Jeong et al reported an esophoric shift in their study which assessed deviation in children with hyperopia and esotropia after the administration of cycloplegia with 0.5% tropicamide and 0.5% phenylephrine mixed eye drops.^[9]^ Their study was retrospectively conducted only on esotropic children; moreover, it did not assess distance-angle deviations and AC/A changes. Given the differences between the present study and Jeong's research, it is not possible to accurately compare the results. Jeong et al also reported that 38% of participants demonstrated esotropic shift (at least 10 prisms) in cycloplegic conditions in near distance, and all of these cases had accommodative esotropia.

Some reasons could be hypothesized as the causes of esophoric shift in deviation. One major reason noted in Jeong's study is that cycloplegia-induced blurred vision is known to be the cause of fusion divergence weakness. This has been reported to give rise to the limited control of esophoria and its subsequent increase. However, this hypothesis probably cannot be true because in present study most of the participants had exophoria. We should note that blurring of vision causes weakness in compensatory fusion. Compensatory fusion in exophoria is convergence and not divergence. If this fusion weakness had been the cause of deviation change, compensatory fusion weakness (convergence weakening in exophoria) should have resulted in an exophoric shift^[11,12]^ rather than an esophoric shift.

The most probable hypothesis to justify the esophoric shift in cycloplegic conditions is related to localized accommodative paralysis and its relationship with near triad response. In fact, in cycloplegic conditions, ciliary muscles muscarinic receptors are locally inhibited, and the accommodation function is disrupted in the muscles involved in the accommodation, whereas using drops other than atropine does not tend to cause complete paralysis, ${\;}^{\left[9,13\right]}$ and the accommodative function partially remains. It is conceivable, however, that although the ciliary muscles are paralyzed, stimulus impulses of near triad response are still in action for accommodation, convergence, and miosis. In such a condition, it is likely that the efforts of the central accommodative system to clarify the image may cause an increase in the stimulus impulses and hence stimulating the accommodative convergence causing an esophoric shift in deviation. The assessment of changes in near deviation with +2.5 addition in this study is strong evidence in support of this hypothesis as compared to the cycloplegic conditions prior to the +2.5 addition which illustrates the lack of need for accommodative effort. As stated in the results, the near deviation has a difference of 11.47 prism diopters with and without cycloplegia. However, when near deviation in cycloplegic conditions is evaluated with a +2.5 addition, the difference with pre-cycloplegic conditions will be only 1.8 prism diopters, which is negligible. Therefore, it can be concluded that the accommodative effort will be zero by the application of 2.5 addition (to the distance correction) for a target at 40 cm, and consequently, no excitatory impulse is released from the brain. In fact, it can be said that blur is driving the additional esodeviation. The lack of impulse emission, in turn, keeps the accommodative convergence system intact, and the near deviation remains approximately constant. In addition to near deviation changes, distance deviation was also examined in the present study. The observed changes in this deviation were < 1 prism diopter, which is clinically insignificant although it is statistically significant. This also reinforces the hypothesis of increased impulse since there is a need for accommodation in near vision, however, there is no accommodative effort for distance (in glasses-corrected conditions); therefore, no significant esophoric shift occurred.

Besides the deviation changes, the results of the present study pointed to changes in the AC/A ratio, and this ratio showed a significant increase in cycloplegic conditions. In fact, despite a significant esophoric shift in near deviation, cycloplegic conditions do not cause a tangible change in distance deviation, and tends to increase the D ${\;}_{n}$ –D ${\;}_{f}$ subtraction, which in turn increases the AC/A ratio. The main limitation of our study was the small sample size secondary to the special circumstances of COVID-19 and the conditions of the study.

In summary, based on the results of the present study, it can be stated that cyclopentolate-induced cycloplegia causes a significant shift in near deviation, whereas distance deviation does not undergo substantial changes. The unequal shift of near and distance deviation also significantly increases the AC/A ratio. Ocular refraction and the magnitude of near deviation are the main factors contributing to cycloplegia-induced esophoric shift.

##  Financial Support and Sponsorship

This project was supported by Iran University of Medical Sciences by the grant number of 98-4-99-16536.

##  Conflicts of Interest

None.
